# Rapid duplex flap probe-based isothermal assay to identify the *Cryptococcus neoformans* and *Cryptococcus gattii*

**DOI:** 10.3389/fcimb.2024.1321886

**Published:** 2024-03-15

**Authors:** Xin Ye, Lei Zhang, Qingqing Yang, Weihua Pan, Xiaoyan Zeng

**Affiliations:** ^1^ Department of Laboratory Medicine, The First Affiliated Hospital of Xi’an Jiaotong University, Xi’an, Shaanxi, China; ^2^ Department of Dermatology, The third affiliated hospital of Xi’an Jiaotong University, Shaanxi Provincial People’s Hospital, Xi’an, Shaanxi, China; ^3^ Department of Dermatology, Shanghai Changzheng Hospital, Naval Medical University, Shanghai, China

**Keywords:** *Cryptococcus neoformans*, *Cryptococcus gattii*, isothermal amplification, flap probe, clinical detection

## Abstract

Cryptococcosis is a life-threatening invasive fungal infection with significantly increasing mortality worldwide, which is mainly caused by *Cryptococcus neoformans* and *Cryptococcus gattii*. These two species complexes have different epidemiological and clinical characteristics, indicating the importance of accurate differential diagnosis. However, the clinically used culture method and cryptococcal capsular antigen detection couldn’t achieve the above goals. Herein, we established a novel duplex flap probe-based isothermal assay to identify the *Cryptococcus neoformans* and *Cryptococcus gattii* within 1 hour. This assay combined the highly sensitive nucleic acid isothermal amplification and highly specific fluorescence probe method, which could effectively distinguish the sequence differences of the two species complexes using two different fluorescence flap probes in a single reaction system. This novel method showed excellent detection performance with sensitivity (10 copies/μL each) and specificity (100%) compared to traditional culture and sequencing methods. Furthermore, we applied this method to spiked clinical samples, 30 cerebrospinal fluids and 30 bronchoalveolar lavage fluids, which kept good detection performance. This novel rapid duplex flap probe-based isothermal assay is a promising and robust tool for applications in differential diagnosis of the *Cryptococcus neoformans* and *Cryptococcus gattii* in clinical settings, especially when clinical suspicion for cryptococcal disease is high and epidemiological studies.

## 1 Introduction

Cryptococcosis is a fatal fungal disease caused only by *Cryptococcus neoformans* and *Cryptococcus gattii* and is estimated to affect one million people every year. The common clinical manifestations are cryptococcal pneumonia or cryptococcal meningitis; the latter is the most common cause of meningitis in people with AIDS, and the mortality rate can be as high as 70% (Rajasingham et al., 2017; Loyse et al., 2019). In October 2022, World Health Organization (WHO) listed *Cryptococcus neoformans* in the Critical Priority Group in its first list of fungi that threaten health, further emphasizing the importance of strengthening clinical and basic research on Cryptococcus (Fisher and Denning, 2023). Although the clinical symptoms of the two species complexes (*Cryptococcus neoformans* and *Cryptococcus gattii*) are similar, their ecology and epidemiology are considered to be different. For example, *C. neoformans* mainly causes infections in immunosuppressed people, while *C. gattii* is more likely to cause infections in immunocompetent people (Baddley et al., 2021; Donnelly et al., 2020). Given the seriousness and importance of cryptococcosis, there is an urgent need for methods that can conveniently, quickly, and accurately identify and differentiate between the two cryptococcal species complexes (*C. neoformans* and *C. gattii*).

Currently commonly used clinical methods for cryptococcal detection include India’s ink method, culture, and cryptococcal capsular antigen (CrAg) detection. The India’s ink method has high specificity and the sensitivity is about 85%, but it relies heavily on the experience of the testing personnel (Bicanic and Harrison, 2004). The culture method is the tool for accurate species complex identification; it also has extremely high specificity and the global sensitivity is up to 95%. However, it is time-consuming, has complex steps, and has many influencing factors. The CrAg test has high sensitivity and specificity, especially when detecting central nervous system and bloodstream infections, but its detection performance is not satisfactory when detecting pulmonary infections (Hsiao et al., 2022). Emerging molecular diagnostic methods, such as sequencing (Zhou et al., 2022) and PCR (Gago et al., 2014), provide effective tools for identifying two types of cryptococci, but sequencing is currently expensive and unsuitable for routine tests. PCR is time-consuming and has high environmental and equipment requirements, which require an independent sample preparation area, amplification area, product analysis area, and an expensive thermal cycler. It is difficult to carry out in areas with limited resources (usually areas with a high incidence of cryptococcosis).

In recent years, the continuous development of isothermal nucleic acid amplification technology has provided innovative tools for rapid and convenient pathogen identification. Isothermal nucleic acid amplification reaction is fast and carried out under constant temperature conditions, which largely overcomes the disadvantages of PCR technology. The isothermal nucleic acid amplification methods that are considered classic and widely used include loop-mediated isothermal amplification, recombinase polymerase amplification, strand displacement amplification, etc (Zhao et al., 2015; De Felice et al., 2022). However, the long length and high concentration of primers employed in isothermal nucleic acid amplification technology can easily lead to false positive signals, thereby restricting its clinical application (Zheng et al., 2023; Wang et al., 2015; Meagher et al., 2018). In our preliminary research, we successfully achieved highly specific and sequence-dependent isothermal nucleic acid amplification by introducing the Flap Endonuclease 1 (FEN1) enzyme and transforming traditional isothermal amplification primers into flap structures, which can be specifically recognized by the FEN1 enzyme (Ye et al., 2019a; Ye et al., 2019b). However, this method is limited to single-plex detection and is unsuitable for multiplex amplification, posing challenges in meeting clinical demands for accurate identification and differentiation of *C. neoformans* and *C. gattii*. Building upon this groundwork, we have developed an innovative flap probe-based isothermal assay by designing two flap structural probes specifically targeting the sequences of *C. neoformans* and *C. gattii*, which are combined with traditional isothermal amplification forward and backward primers. This technology has successfully demonstrated its efficiency in identifying and differentiating *C. neoformans* and *C. gattii* in two common clinical sample types (cerebrospinal fluid and bronchoalveolar lavage fluid).

## 2 Materials and methods

### 2.1 Regents and equipment

**Figure 1:**
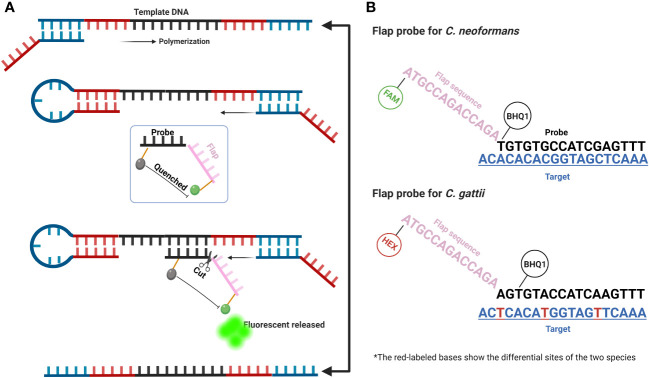
Schematic diagram of FPIA method. **(A)** A simplified diagram of the amplification steps of the FPIA method; **(B)** Structure of the flap probe of *C. neoformans* and *C. gattii*.

Plasmids containing conserved gene sequences of *C. neoformans* and *C. gattii* were separately synthesized (Shanghai Sangon Biotech). The sequences of the flap-probe primers (fluorescent and quencher labeled), forward and reverse primers (Shanghai Sangon Biotech) used in this study are listed in **Table 1**. The forward and reverse primers were generated using inner primers’ design method within loop-mediated isothermal amplification (https://primerexplorer.jp/e/), composed of F1c, F2, and B1c, B2, respectively. The flap-probe primer consists of two parts: the recognition region that matches the target sequence and the flap region that does not match the target sequence at all (shown in **Figure 1**).

**Table 1:** The sequences of flap-probe primers, forward and reverse primers used in this study (the bases marked in red are the flap region, which is a mismatch sequence that does not match the template at all).

Item	Sequences (5’-3’)
Forward	GTACTAGAGCTAGAGCTGCTAGAATAACTGTTAATAATGCTACACTTAACCG
Reverse	CCTGAAGGTATTAGCTCAAATGCGATCCTTAAAGATAAAGTATGGATGC
Flap-probe primer for *C. neoformans*	FAM-ATGCCAGACCAGA/iBHQ1dT/GTGTGCCATCGAGTTT
Flap-probe primer for *C. gattii*	HEX-ATGCCAGACCAGAAG/iBHQ1dT/GTACCATCAAGTTT

The *Bst* DNA polymerases, large fragment (M0275) along with the supplementary reaction buffers and the thermostable flap endonuclease, FEN1 (M0645), were purchased from New England Biolabs. The fungi genomic DNA extraction kit (D2300) was purchased from Beijing Solarbio. The detection of fluorescent signals during the amplification was based on the SLAN-96P PCR system (Shanghai Hongshi Medical Technology). The Tt value, defined as the time to reach the fluorescence threshold during amplification, was automatically determined by the PCR system software.

### 2.2 Clinical samples collection and processing

A total of 13 C*. neoformans* strains and 7 C*. gattii* strains were used in this study. They were collected from the First Affiliated Hospital of Xi’an Jiaotong University and Shanghai Changzheng Hospital, and some were purchased from Beijing Beina Chuanglian Biotech Institute. The common lineages within each species complex were included in this study using the ATCC strains. The exact information of these strains were shown in **Supplementary Table S1**. In addition, 2 *Candida albicans*, 2 *Candida tropicalis*, 2 *Candida parapsilosis*, 2 *Candida glabrata* and 2 *Candida krusei* were collected from the First Affiliated Hospital of Xi’an Jiaotong University, which served as negative controls. All above strains were sequenced and identified correctly. All above strains were recovered, and the genomic DNA was extracted following the manufacturer’s instructions (Roche KAPA Express Extract kit), which could be completed in 15 minutes. The extracted DNA was partitioned and stored at -80°C until use.

### 2.3 The reaction system of duplex flap probe-based isothermal nucleic acid assay (FPIA)

The total volume of the duplex FPIA was 25 μL. The final concentration of each composition of the reaction system is as follows: 1.6 μM each for forward and reverse primers, 1 μM of flap-probe each for *C. neoformans* and *C. gattii*, 1×ThermoPol Buffer (B0537, New England Biolabs), 6 mM MgSO4, 1.4 mM dNTP mix (each), 8 U *Bst* DNA polymerase, 1.2 U FEN1, 2.5 μL nucleic acid template, and UltraPure water.

First, only one flap probe was added to the system to verify the detection ability targeting the single species complexes. Then, the reaction was performed at 60°C and 65°C, respectively, to assess the best temperature conditions. Finally, two flap probes were added to verify the duplex FPIA targeting the two species complexes.

### 2.4 Detection performance evaluation of duplex FPIA

The DNA sequences of multiple evolutionary divergent lineages of the two species complexes were downloaded from the public database (NCBI). The sequence alignment results, and the locations of the primers and probes were shown in **Supplementary Figure S1** and **S2**.

The known concentrations of plasmid samples carrying the highly conserved DNA sequences of the two species complexes were serially diluted (concentration range was 10^6^-10^1^ copies/μL), and the gradient mentioned above dilution samples were tested using a duplex FPIA method (each concentration was repeated three times) to evaluate the sensitivity of the detection.

Next, the linear relationship between the average Tt value from three replicate tests for each gradient concentration and the logarithm value of the corresponding concentration was analyzed to evaluate the semi-quantitative capability of the new method.

Finally, a variety of samples containing other common infecting fungi, such as *C. albicans*, *C. tropicalis*, *C. parapsilosis*, *C. glabrata* and *C. krusei* were utilized to evaluate the specificity of the novel FPIA method.

### 2.5 Evaluation in clinical applications

To assess the clinical applicability, spiked clinical samples were utilized. A total of 30 clinically determined uninfected cerebrospinal fluids (CF) and bronchoalveolar lavage fluids (BALF) were collected from the First Affiliated Hospital of Xi’an Jiaotong University, respectively. These two sample types are critical for the diagnosis of the two major cryptococcal diseases, cryptococcal meningitis and cryptococcal pneumonia. All strains mentioned above were recovered and added to the CFs and BALFs, which created the spiked clinical samples and were used to evaluate the detection performance of the present novel method. The nucleic acids of the above spiked clinical samples were extracted following the manufacturer’s instructions and stored at -80°C until use.

It is important to note that all samples were collected retrospectively and had no bearing on clinical decision-making or treatment outcomes for the patients. Therefore, the patient’s informed consent was exempted from the ethics committee of the First Affiliated Hospital of Xi’an Jiaotong University.

## 3 Results

### 3.1 The mechanism of the novel duplex flap probe-based isothermal nucleic acid assay (FPIA)

**Figure 1A** shows the composition and amplification steps of the FPIA system. Specifically, the FPIA system includes a pair of forward and reverse primers and a flap probe. Similar to previous studies, under the combined action of *Bst* DNA polymerase, forward and reverse primers, the primary product of the neck loop structure at both ends can be rapidly polymerized. At this time, the probe sequence in the flap probe structure will search the sequence in the target, which is completely paired with it, and forms a flap structure with the target sequence that can be specifically recognized by the FEN1 enzyme. Then, the cleavage activity of FEN1 enzyme will be activated, and the flap structure on the flap probe will be cut off, so that the labeled fluorescence and quenching group will be separated, and the fluorescent signal will be released. **Figure 1B** further shows the differences between the sequences of *C. neoformans* and *C. gattii*, as well as the different flap probe sequences of the two species complexes. The two flap probes are labeled with different fluorescent groups (FAM and HEX). The duplex FPIA detection mode can determine which species complexes it is based on the different colors of the fluorescence signals. The target sequences of the two species complexes have multiple base differences. By designing two different flap probes, they can specifically recognize their target sequences respectively, ensuring the specificity of the reaction.

### 3.2 The feasibility verification of duplex FPIA

**Figure 2:**
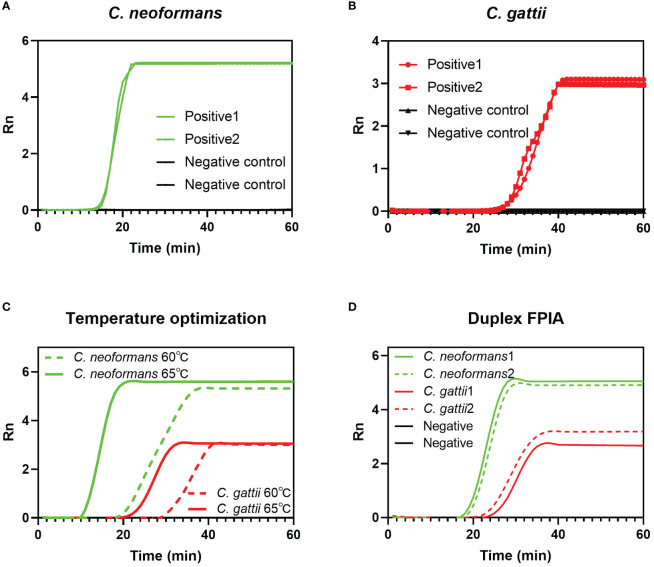
Feasibility verification of FPIA method. **(A)** Single-plex FPIA to detect *C. neoformans*; **(B)** Single-plex FPIA to detect *C. gattii*; **(C)** FPIA reaction temperature optimization; **(D)** Duplex FPIA to detect *C. neoformans* and *C. gattii* simultaneously.

**Figures 2A** and **B** respectively show the efficiency of the single-plex FPIA method in detecting nucleic acids of two species complexes, *C. neoformans* and *C. gattii*. The results show that when the FPIA system contains only one flap probe, the target can be successfully detected, and negative controls (ultrapure water) maintain a negative signal. The results in **Figure 2C** demonstrate that the detection of both species complexes at 65°C is better than at 60°C, which have smaller Tt values. **Figure 2D** demonstrates the detection capability of duplex FPIA. When there are two flap probes for the two species complexes coexisted in the FPIA system (labeled with FAM fluorescence and HEX fluorescence groups, respectively), the target can still be accurately detected. When the *C. neoformans* sequence is present, a green fluorescence signal (FAM) appears, and when the *C. gattii* sequence is present, a red fluorescence signal (HEX) appears.

### 3.3 Detection performance of duplex FPIA in identifying and distinguishing the *C. neoformans* and *C. gattii*

**Figure 3:**
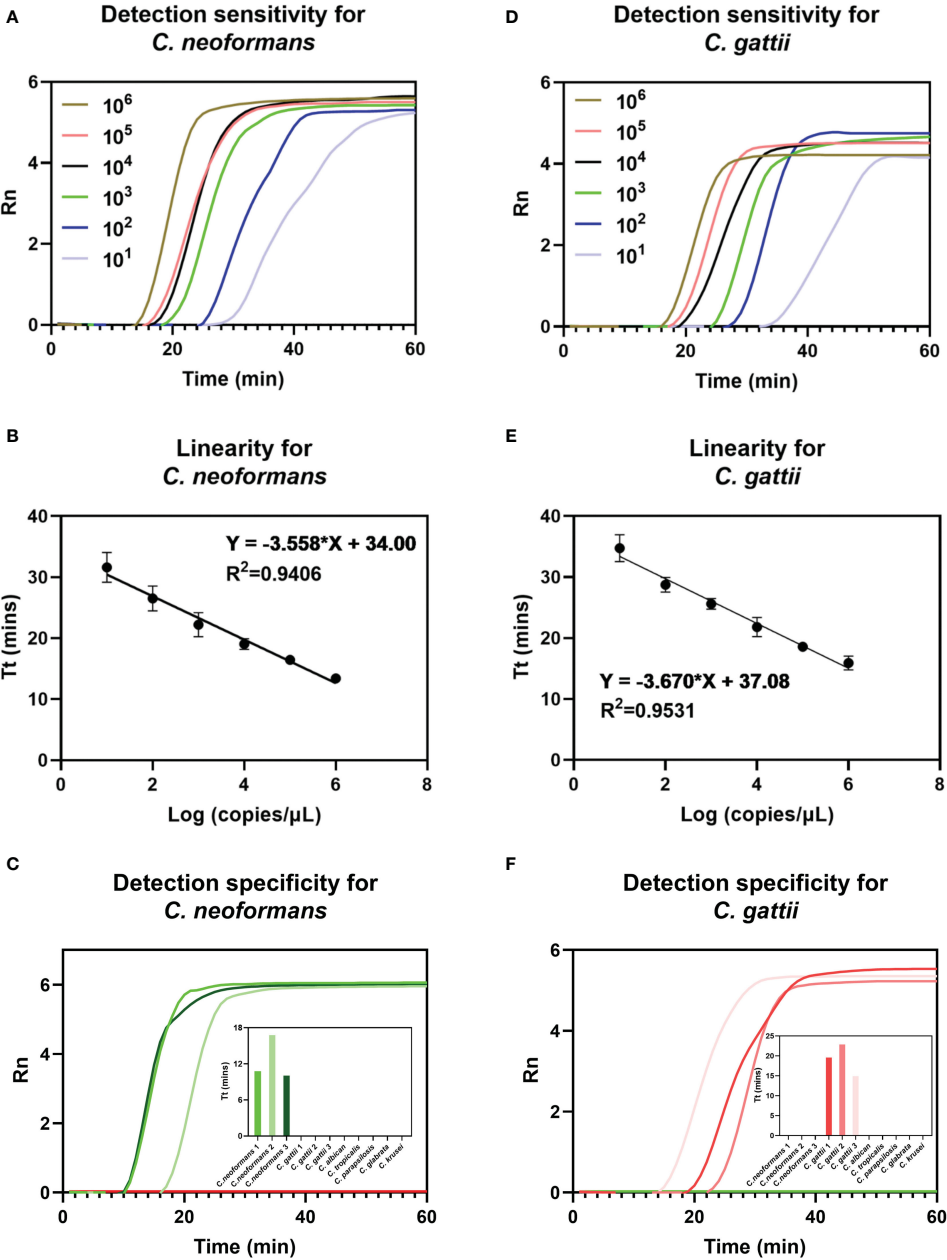
Detection performance evaluation of duplex FPIA method. The detection sensitivity **(A)**, linearity **(B)** and specificity **(C)** for *C. neoformans.* The detection sensitivity **(D)**, linearity **(E)** and specificity **(F)** for *C. gattii*.

**Figures 3A** and **D** show that the sensitivity of duplex FPIA for detecting *C. neoformans* and *C. gattii* can reach 10^1^copies/μL, respectively. **Figures 3B** and **E** show that there is a good linear relationship (*p*<0.0001) between the mean Tt values of repeated detections for each concentration gradient of the two species complexes and the logarithm of their corresponding concentrations. **Figures 3C** and **F** further demonstrate the excellent specificity of the duplex FPIA assay. In the presence of other common infecting yeasts as controls, only when *C. neoformans* and *C. gattii* are present do the corresponding fluorescent signals emerge, underscoring the assay’s ability to discern the target species with high specificity.

### 3.4 Detection performance of duplex FPIA in spiked clinical samples

**Figure 4:**
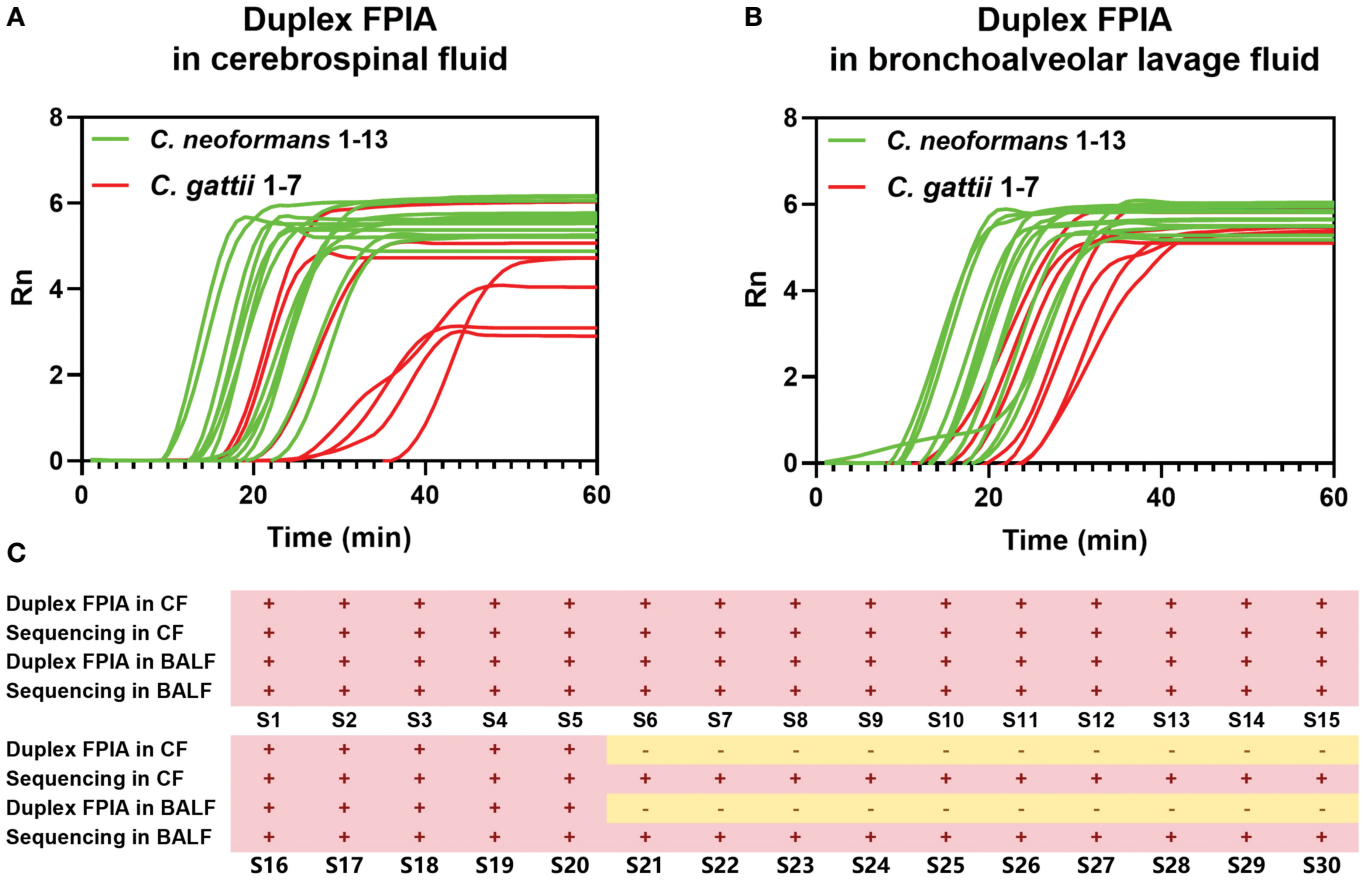
Detection performance evaluation of duplex FPIA method in spiked clinical samples. **(A)** The detection performance in cerebrospinal fluids; **(B)** The detection performance in bronchoalveolar lavage fluids; **(C)** Comparison of duplex FPIA test results and sequencing results.

**Figures 4A** and **B** underscore the precision of the duplex FPIA in accurately detecting the presence of *C. neoformans* and *C. gattii* infections in cerebrospinal fluids and bronchoalveolar lavage fluids, respectively. Furthermore, the results in **Figure 4C** affirm that the detection outcomes of the duplex FPIA align perfectly and are 100% consistent with the strain sequencing identification results.

## 4 Discussion

Cryptococcosis, a pervasive fungal infection, exhibits a global distribution, affecting both immunosuppressed and immunocompetent people. The incidence is particularly high in South Africa and Asia regions which covers most resource-limited areas in the world (Beardsley et al., 2019; Sloan and Parris, 2014). *C. neoformans* and *C. gattii* are the main pathogens causing cryptococcosis, which has different epidemiological and clinical characteristics. Traditional methods have different disadvantages in the diagnosis and identification of both species complexes. Especially in areas with limited resources, many experimental technologies (such as sequencing, mass spectrometry, etc.) are difficult to carry out. Therefore, a convenient, fast, and accurate method for diagnosing and identifying the two species complexes is highly needed.

The duplex FPIA method presented in this study aptly addresses the aforementioned diagnostic challenges. Notably, the method responds quickly (completed within 1 hour) and can effectively identify *C. neoformans* and *C. gattii* simultaneously (offering satisfactory sensitivity and specificity). The entire reaction is conducted under constant temperature conditions, ensuring methodical convenience—a notable improvement over traditional methods such as PCR (Zeller et al., 2017; Trubiano et al., 2016). The FPIA method integrates the efficiency of nucleic acid amplification guided by forward and reverse primers under the action of *Bst* DNA polymerase. Simultaneously, it incorporates sequence-dependent, specific fluorescence signal generation guided by the FEN1 enzyme and flap probe. This amalgamation not only leverages the high efficiency characteristic of traditional isothermal nucleic acid amplification methods but also enhances specificity through signal generation modifications, particularly advantageous in multiplex detection scenarios (Zhao et al., 2018).

The versatility of the FPIA method extends to its applicability in both single-plex and duplex modes, enhancing its utility compared to previous research (Tian et al., 2022), this flexibility allows users to select the mode aligning with their specific detection objectives. In the duplex detection mode, its sensitivity for detecting both *C. neoformans* and *C. gattii* can reach 10^1^copies/μL, which avoids missed detection to the greatest extent and enables accurate identification of the two species complexes within a single-tube reaction.

The robust linear relationship observed between the detection concentration and Tt value also suggests that the FPIA method has potential semi-quantitative capabilities. The duplex FPIA test maintains excellent specificity when detecting *C. neoformans*, *C. gattii* and other common infectious yeasts. This high degree of specificity ensures that there will be no clinical misdiagnosis. When processing spiked clinical samples (CFs and BALFs), the duplex CFPA method also maintained excellent detection performance, and its identification results were 100% consistent with the sequencing identification results.

While this study contributes valuable insights, certain limitations should be acknowledged. First, spiked clinical samples made from pure culture colonies were used to evaluate the detection performance, which may be different from real infected clinical samples. Second, this study collected samples retrospectively and the number of samples is limited. In the future, more real clinical infection samples need to be prospectively collected to further evaluate the clinical detection performance of duplex FPIA.

In summary, our study establishes a novel FPIA method designed for the rapid, convenient, and accurate identification of *C. neoformans*, *C. gattii* in different clinical samples (CFs and BALFs). The method exhibits a high level of agreement with culture and sequencing results. This novel approach is poised to significantly contribute to the precise differential diagnosis of clinical cryptococcosis pathogens, thereby improving patient prognosis. Moreover, it presents a valuable tool for ecological and epidemiological research on cryptococcosis.

## Data availability statement

The original contributions presented in the study are included in the article/**Supplementary Material**. Further inquiries can be directed to the corresponding authors.

## Ethics statement

The studies involving humans were approved by The ethics committee of the First Affiliated Hospital of Xi’an Jiaotong University. The studies were conducted in accordance with the local legislation and institutional requirements. The human samples used in this study were acquired from a by-product of routine care or industry. Written informed consent for participation was not required from the participants or the participants’ legal guardians/next of kin in accordance with the national legislation and institutional requirements.

## Author contributions

XY: Conceptualization, Formal Analysis, Funding acquisition, Investigation, Methodology, Writing – original draft. LZ: Funding acquisition, Software, Validation, Writing – original draft. QY: Methodology, Resources, Validation, Writing – original draft. WP: Investigation, Resources, Writing – review & editing. XZ: Funding acquisition, Project administration, Resources, Supervision, Writing – review & editing.
