# Supplementary material for: Rapid duplex flap probe-based isothermal assay to identify the Cryptococcus neoformans and Cryptococcus gattii
Source: Front Cell Infect Microbiol. 2024 Mar 15;14:1321886. doi: 10.3389/fcimb.2024.1321886 (PMC10981274; doi:10.3389/fcimb.2024.1321886)
Supplement: Supplementary file 1 [file DataSheet_1.docx]

**Supporting information**

Rapid duplex flap probe-based isothermal assay to identify the *Cryptococcus neoformans* and *Cryptococcus gattii*

Xin Ye^1†^, Lei Zhang^2†^, Qingqing Yang^1^, Weihua Pan^3^, Xiaoyan Zeng^1*^

1. Department of Laboratory Medicine, The First Affiliated Hospital of Xi’an Jiaotong University, Xi’an 710061, Shaanxi, PR China.
2. Department of Dermatology, The third affiliated hospital of Xi'an Jiaotong University, Shaanxi Provincial People's Hospital, Xi’an 710061, Shaanxi, PR China.
3. Department of Dermatology, Shanghai Changzheng Hospital, Naval Medical University, Shanghai 200003, PR China.

† Xin Ye and Lei Zhang contributed equally to this work.

* To whom correspondence should be addressed. Dr. Xiaoyan Zeng, Tel: 86-29-85324076; Email: xiaoyanzeng@xjtu.edu.cn

Supplementary Materials and Methods

The target gene is mitochondrial cytb gene. The sequence alignments of multiple lineages within the two species complex and the locations of the primers and probes were shown in Figure S1 and Figure S2 respectively. The sequence alignment is performed by Vector NTI 11.5.0.


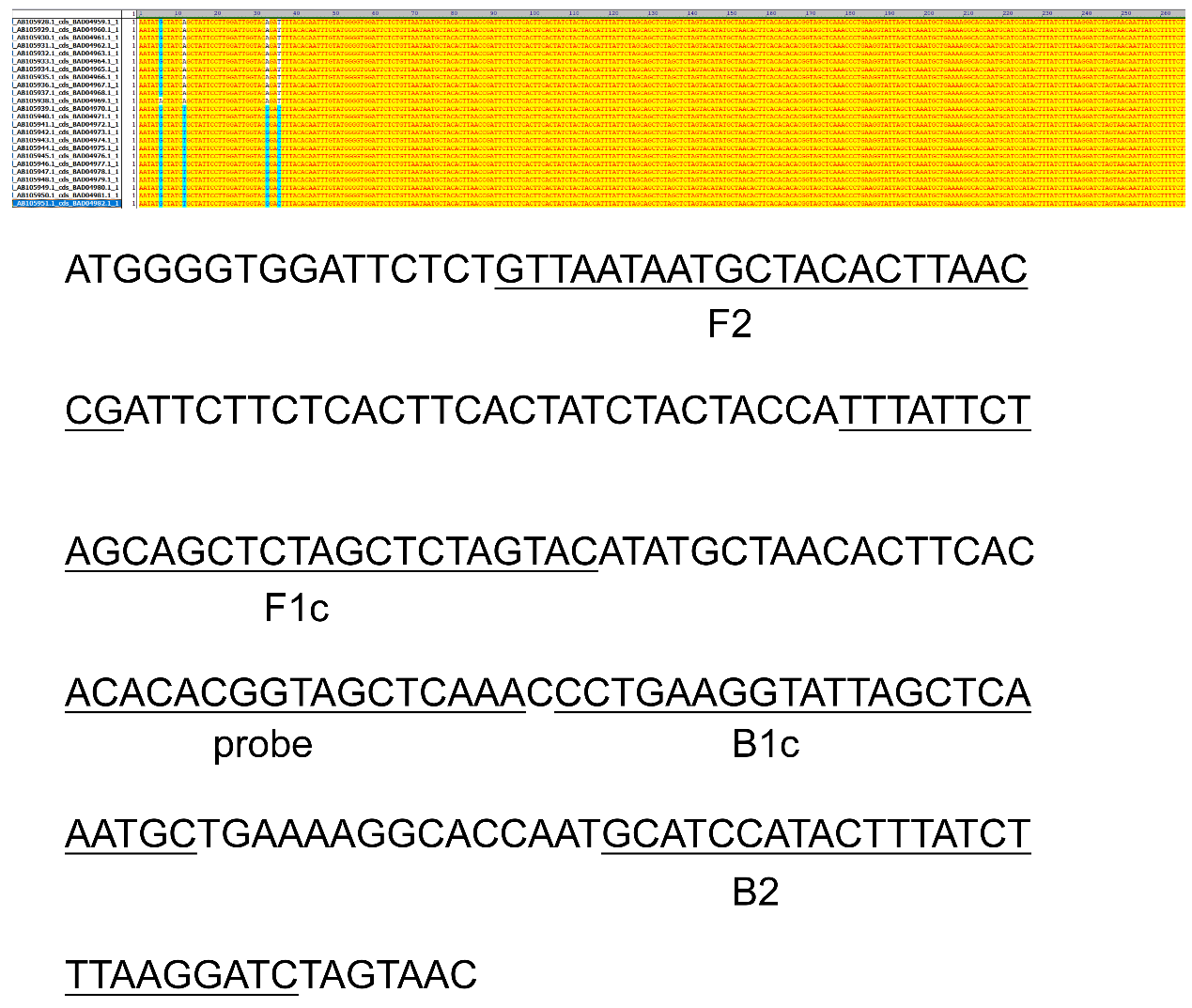


Figure S1 The sequence alignment and the locations of the primers and probes of the *C. neoformans* species complex. The strains included in the alignment are IFM 48638, IFM 46734, IFM 46729, IFM 46572, IFM 46554, IFM 46132, IFM 45756, IFM 45737, IFM 45708, IFM 5854, IFM 5506, IFM 5505, IFM 5889, IFM 48643, IFM 48642, IFM 48641, IFM 48640, IFM 46090, IFM 46082, IFM 5881, IFM 5857, IFM 5845, IFM 5844, IFM 48639.


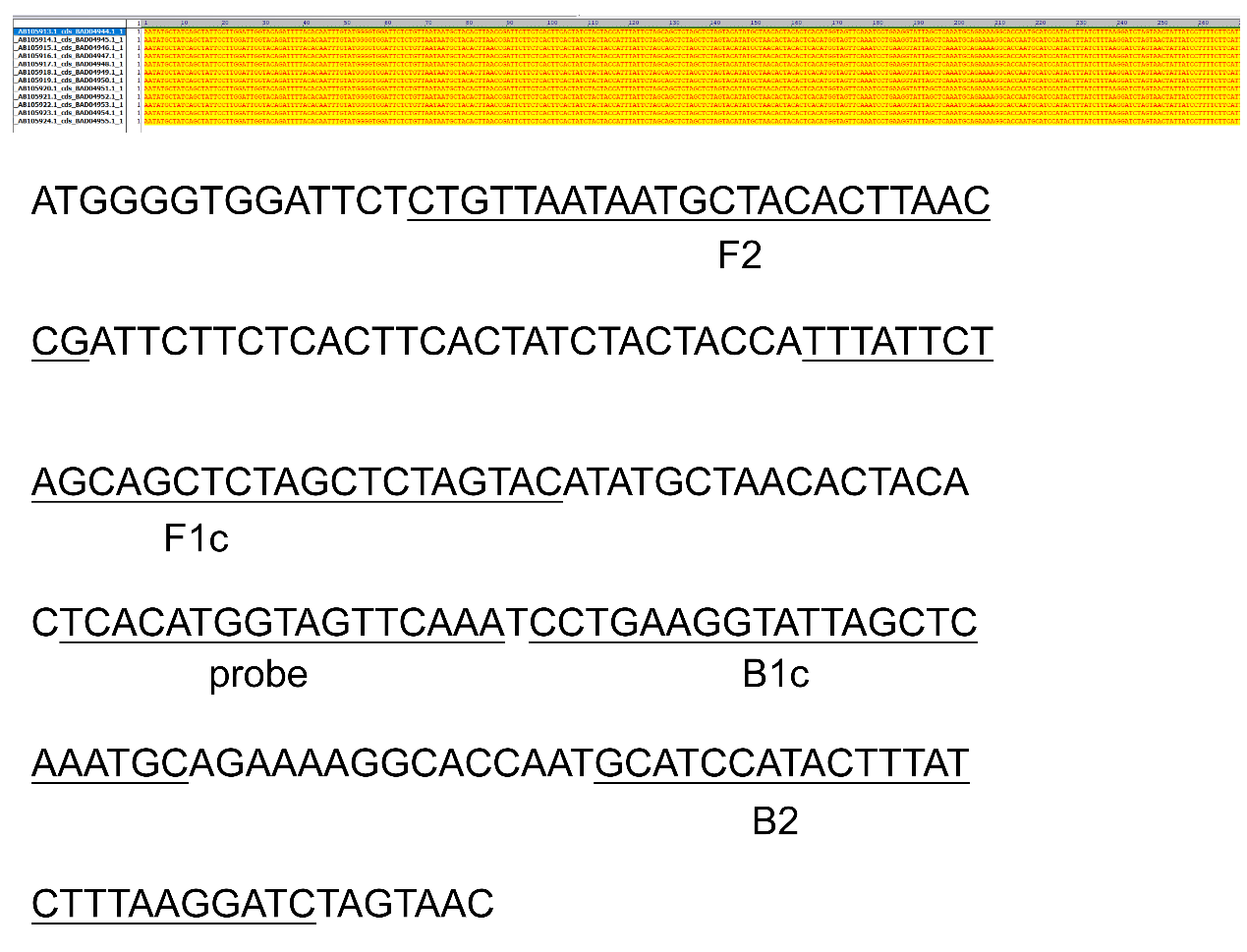


Figure S2 The sequence alignment and the locations of the primers and probes of the *C.* *gattii* species complex. The strains included in the alignment are IFM 5815 (VGⅠ), IFM 48634 (VGⅠ), IFM 48636 (VGⅠ), IFM 48819 (VGⅠ), IFM 48820 (VGⅠ), IFM 48821 (VGⅠ), IFM 5856 (VGⅠ), IFM 5873 (VGⅠ), IFM 5875 (VGⅠ), IFM 5878 (VGⅠ), IFM 48635 (VGⅠ), IFM 5855 (VGⅠ).

Table S1. The DNA extracts of the strains belonging the major gene type of the two species complexes used in this study.

| Gene type | Strain |
| --- | --- |
| VNⅠ | ATCC 208821 |
| VNⅡ | ATCC MYA4565 |
| VNⅢ | ATCC MYA 4566 |
| VNⅣ | ATCC MYA 4567 |
| VGⅠ | ATCC MYA4560 |
| VGⅡ | ATCC MYA 4561 |
| VGⅢ | ATCC MYA 4562 |
| VGⅣ | ATCC MYA 4563 |
